# Genome-Wide Expression in Visceral Adipose Tissue from Obese Prepubertal Children

**DOI:** 10.3390/ijms16047723

**Published:** 2015-04-08

**Authors:** Concepción M. Aguilera, Carolina Gomez-Llorente, Inés Tofe, Mercedes Gil-Campos, Ramón Cañete, Ángel Gil

**Affiliations:** 1 Department of Biochemistry and Molecular Biology II, Institute of Nutrition and Food Technology, Centre for Biomedical Research, University of Granada, Armilla, 18100 Granada, Spain; E-Mails: caguiler@ugr.es (C.M.A.); gomezll@ugr.es (C.G.-L.); 2 Unit of Pediatric Endocrinology, Reina Sofia University Hospital, Avda Menéndez Pidal s/n. 14004 Córdoba, Spain; E-Mails: drakaramelo@hotmail.com (I.T.); mercedes_gil_campos@yahoo.es (M.G.-C.); em1caesr@uco.es (R.C.)

**Keywords:** obesity, visceral adipose tissue, prepubertal children, microarray

## Abstract

Characterization of the genes expressed in adipose tissue (AT) is key to understanding the pathogenesis of obesity and to developing treatments for this condition. Our objective was to compare the gene expression in visceral AT (VAT) between obese and normal-weight prepubertal children. A total of fifteen obese and sixteen normal-weight children undergoing abdominal elective surgery were selected. RNA was extracted from VAT biopsies. Microarray experiments were independently performed for each sample (six obese and five normal-weight samples). Validation by quantitative PCR (qPCR) was performed on an additional 10 obese and 10 normal-weight VAT samples. Of 1276 differentially expressed genes (*p* < 0.05), 245 were more than two-fold higher in obese children than in normal-weight children. As validated by qPCR, expression was upregulated in genes involved in lipid and amino acid metabolism (*CES1*, *NPRR3* and *BHMT2*), oxidative stress and extracellular matrix regulation (*TNMD* and *NQO1*), adipogenesis (*CRYAB* and *AFF1*) and inflammation (*ANXA1*); by contrast, only *CALCRL* gene expression was confirmed to be downregulated. In conclusion, this study in prepubertal children demonstrates the up- and down-regulation of genes that encode molecules that were previously proposed to influence the pathogenesis of adulthood obesity, as well as previously unreported dysregulated genes that may be candidate genes in the aetiology of obesity.

## 1. Introduction

Childhood obesity is one of the most important public health problems of the 21st century, affecting both developed and developing countries. Early-onset obesity is associated with an increased incidence of adult obesity, type 2 diabetes, non-alcoholic fatty liver disease and cardiovascular risks factors [1].

Obesity is characterized by an increased body mass index (BMI) as a consequence of weight gain, which may be implicated in the remodelling of adipose tissue (AT) [2]. Currently, it is known that AT acts as an endocrine organ that is capable of secreting hormones, cytokines, growth factors and vasoactive substances, among other factors, which are involved in regulating body weight, glucose homeostasis, lipid metabolism angiogenesis, reproduction and immunity [3,4]. AT can be divided into specific depots; subcutaneous adipose tissue (SAT) and visceral adipose tissue (VAT) are two major types of AT in adult humans, and they have distinct characteristics [5]. Metabolic disorders are more strongly associated with VAT, which is possibly because secretions from this tissue may reach the liver and because the gene expression profile and the biochemical and metabolic properties of VAT may also contribute to the preferential association of VAT with metabolic dysfunction in obese subjects [6]. Differences in gene expression between SAT and VAT have previously been reported in adults. VAT is associated with insulin resistance, diabetes, hypertension, atherosclerosis, and hepatic steatosis, whereas SAT responds better to insulin and secretes more adiponectin and smaller amounts of inflammatory cytokines [7]. Moreover, VAT from patients with extreme obesity expresses more genes than SAT or epigastric AT, suggesting a more specialized lineage and specific functional properties of this depot [8]. 

Therefore, studying the genome-wide mRNA expression profile of VAT might be a useful tool for analysing the mechanisms of weight regulation and understanding the development of obesity and its comorbidities. Most microarray-based expression studies that have been published to date have been performed on obese adults and have compared the gene expression profiles in VAT *vs.* SAT [8,9,10]. Only two have examined the different expression profiles in the VAT of obese adults and normal-weight adults [11,12]. Regarding studies performed in children, only one has compared the gene expression data for SAT and VAT [13]. Studies in prepubertal children can offer important insight into the genetic basis of obesity because excess weight during the growing years constitutes a major risk for being overweight or obese during adult life. Furthermore, prepubertal children are not affected by the hormonal changes associated with puberty, which affect body fat distribution [14].

Overall, our primary objective was to determine the gene expression profile of fat depots in obese and normal-weight prepubertal children. To the best of our knowledge, this study is the first to analyse whole genome expression in VAT from prepubertal obese children and to compare it with that in normal-weight children. The present study was performed to clarify the effect of obesity on the gene expression profile of VAT in prepubertal children, by using DNA microarrays and subsequently to validate these findings with quantitative PCR (qPCR) methods.

## 2. Results and Discussion

### 2.1. Anthropometric and Biochemical Characteristics of the Study Population

The clinical characteristics of the 11 children selected for the microarray analysis and those of the 20 additional children included in the qPCR analysis are shown in Table 1.

**Table 1:** Clinical and biochemical characteristics of the prepubertal children.

	Microarray			qPCR
Characteristics	Normal-Weight (*n* = 6)	Obese (*n* = 5)	Normal-Weight (*n* = 10)	Obese (*n* = 10)
Sex (M:F)	5:1	5:0	6:4	8:2
Age (years)	8.2 ± 0.6	10.2 ± 0.6	9.3 ± 0.8	9.5 ± 0.5
Weight (kg)	28.8 ± 1.1	55.8 ± 2.9 *	32.9 ± 2.7	50.9 ± 3.6 *
Height (m)	131.4 ± 3.4	141.0 ± 4.0	136.2 ± 4.2	138.9 ± 4.3
BMI (kg/m^2^)	16.8 ± 0.6	28.1 ± 0.7 *	17.5 ± 0.5	26.1 ± 0.7 *
BMI z score	−0.61 ± 0.36	3.12 ± 0.5 *	−0.6 ± 0.2	2.8 ± 0.3 *
Systolic BP (mm·Hg)	111.3 ± 3.20	123.0 ± 7.1	109.1 ± 2.6	119.9 ± 4.5
Diastolic BP (mm·Hg)	61.3 ± 3.0	71.2 ± 5.6	60.3 ± 3.2	67.8 ± 3.9
Glucose (mmol/L)	4.73 ± 0.30	4.95 ± 0.19	4.9 ± 0.2	5.1 ± 0.3
Insulin (pmol/L)	9.0 ± 1.6	19.3 ± 7.2	10.4 ± 1.6	11.1 ± 1.4
HOMA-IR	1.8 ± 0.32	4.3 ± 1.7	2.3 ± 0.33	2.4 ± 0.31
TAG (mmol/L)	0.77 ± 0.19	0.67 ± 0.09	0.88 ± 0.18	0.80 ± 0.09
TC (mmol/L)	4.19 ± 3.44	4.21 ± 0.57	3.86 ± 0.25	4.22 ± 0.42
HDL-C (mmol/L)	1.54 ± 0.09	1.78 ± 0.23	1.42 ± 0.15	1.40 ± 0.11
LDL-C (mmol/L)	2.29 ± 0.26	2.11 ± 0.43	2.02 ± 0.21	2.44 ± 0.34
Apo A-1 (g/L)	0.12 ± 0.08	1.44 ± 0.05	1.23 ± 0.10	1.19 ± 0.09
Apo B (g/L)	0.74 ± 0.09	0.61 ± 0.10	0.67 ± 0.07	0.73 ± 0.09
Adiponectin (mg/L)	14.8 ± 0.3	5.9 ± 3.6	16.7 ± 1.9	11.8 ± 1.9
Leptin (μg/L)	27.9 ± 2.3	33.1 ± 4.8	27.3 ± 1.7	34.8 ± 3.4
CRP (g/L)	0.29 ± 0.09	0.32 ± 0.14	0.29 ± 0.08	0.46 ± 0.09

M, male; F, female; BP, blood pressure; HOMA-IR, homeostasis model assessment of insulin resistance; TAG, triacylglycerols; TC, total cholesterol; HDL-C, HDL-cholesterol; LDL-C, LDL-cholesterol; Apo, apolipoprotein; and CRP, C-reactive protein. The data are shown as the means ± SEM. Differences between the normal-weight and obese groups were analysed with the non-parametric Mann-Whitney U test. * *p* < 0.05.

There were no significant differences in the age or height between obese and control children; however, the weight and BMI were significantly higher in the obese subjects included in both the array and qPCR analyses. No significant differences were found in the blood pressure, fasting glucose, insulin, triacylglycerol (TAG), total cholesterol, insulin resistance (calculated by the homeostatic model assessment, HOMA-IR) or lipoprotein-related parameters. The fasting plasma adiponectin concentrations tended to be lower in the obese children than in the normal-weight children in both analyses. Conversely, the leptin plasma level tended to be higher in the obese *vs.* normal-weight children. The C-reactive protein plasma concentrations were similar between the obese and normal-weight selected children for the microarray analysis and for the qPCR confirmation.

There were no significant differences in the carbohydrate and lipid metabolism parameters of normal-weight and obese children, which could be because of the small number of samples. In fact, we described higher systolic and diastolic BP, as well as higher plasma TAG, insulin and HOMA-IR and lower HDL-C levels in a bigger population of study of obese children [15,16].

### 2.2. Gene Expression Profile of Visceral Adipose Tissue in Obese Children

**Figure 1:**
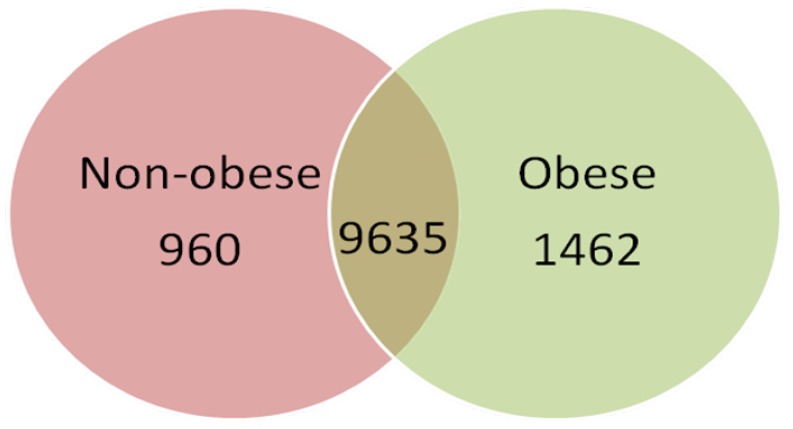
Venn diagram of the expressed sequences in the visceral adipose tissue of obese and normal-weight prepubertal children. After filtering the data with the Affymetrix Detection Algorithm, a total of 12,057 sequences were found to be expressed in adipose tissue. Each condition is represented by a circle in the Venn diagram. The numbers in the region of the overlapping circles indicate the genes that are expressed in both conditions.

**Figure 2:**
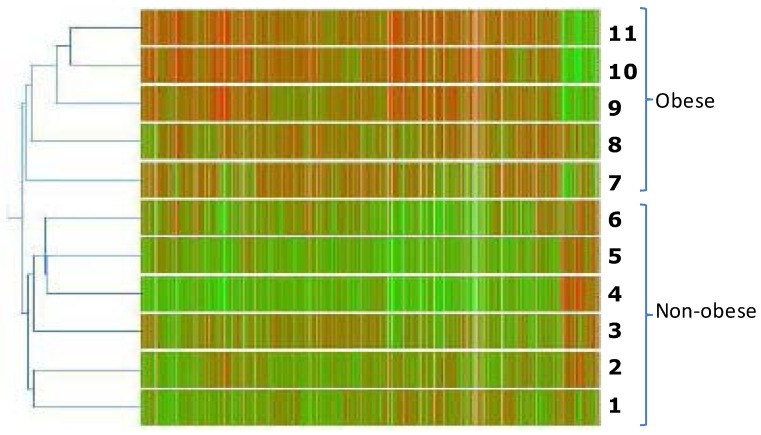
Microarray analysis of the transcripts expressed in the visceral adipose tissue from obese *vs.* normal-weight (control) prepubertal children. RNA from each patient was subjected to chip analysis using Affymetrix Human Genome U133Plus 2.0 GeneChips. The normal-weight group consists of six individuals (numbers 1–6), and the obese group consists of five patients (numbers 7–11). Bidimensional cluster analysis was performed using a Pearson correlation. The parametric test ANOVA was used to uncover the 1588 genes that were differentially expressed.

The gene expression profile of VAT tissue from six control and five obese children was obtained using the Human Genome HG-U133 Plus 2.0 array from Affymetrix. After filtering data with the Affymetrix Detection Algorithm, out of 12,057 filtered sequences, 11,097 were expressed in obese and 10,595 in normal-weight children. The majority of genes were expressed in both conditions; 960 genes were exclusively expressed in tissues from normal-weight children and 1462 in obese adipose (Figure 1). Of the 12,057 filtered sequences, 1588 were differentially expressed between obese children and normal-weight children (*p* < 0.05), as determined by GeneSpring software. Hierarchical clustering analysis of these genes allowed the samples to be adequately separated by experimental condition (obese or normal-weight) (Figure 2).

Out of the 1588 probe sets, 1276 corresponded to single annotated genes because more than one probe set detected a similar change for the same gene in some cases, providing additional validation of the expression data. Out of these 1276 genes, 245 were expressed at a more than two-fold higher level in AT from obese children than in AT from normal-weight children, including 201 that were up-regulated (82%) (Table S1) and 44 that were down-regulated (18.1%) (Table S2). The 245 differentially expressed genes were classified according to the biological process in which they are involved, following the criteria of the Gene Ontology (GO) Consortium. The genes belong to a diverse range of biological processes, including metabolism, cell signalling and cell adhesion, and extracellular matrix development (Tables S1 and S2).

Additionally, differentially expressed transcripts were subjected to Ingenuity Pathway Analysis. “Lipid Metabolism, Small Molecule Biochemistry, Endocrine System, Development and Function” was the top score network identified. Twenty-two of 26 genes involved in the network were upregulated and four were down-regulated (Figure S1). Regarding the canonical pathway, four of them were positive z-score: Gαi Signalling (*p* = 0.0011) with seven molecules of 118 (0.059) (*ADRA2A*↑, *GNAI1*↑, *GNG11*↑, NPR3*↑, *P2RY14*↑, *RGS4*↑, *SHC1*↑), cAMP-mediated signalling (*p* = 0.0012) with 7 molecules of 213 (0.033) (*ADRA2A*↑, *ADRB2*↑, *AKAP12*↑, *GNAI1*↑, NPR3*↑, *P2RY14*↑, *RGS4*↓), ILK signalling (*p* = 0.0365) with six molecules of 181 (0.033) (*ARHGEF6*↑, *CCND1*↑, *IRS2*↑, *ITGB5*↑, *JUN*↑, *RHOB*↑), and IL-8 Signalling (*p* = 0.0382) with six molecules of 183 (0.033) (*CCND1*↑, *GNAI1*↑, *GNG11*↑, *ITGB5*↑, *JUN*↑, *RHOB*↑). No pathway was found to have a negative z-score.

This study is the first to shows a distinct gene expression pattern in VAT from obese and normal-weight prepubertal children. As in adults [11,12], the majority of the differentially expressed genes encoded proteins that are mainly involved in metabolism, cell signalling, immune responses, the extracellular matrix and signal transduction. Moreover, our results also revealed the up-regulation of genes involved in cell death (regulation of apoptosis and regulation of programmed cell death) and cell proliferation regulation, but down-regulation of the genes involved in induction of apoptosis and induction of programmed cell death were also observed. Furthermore, we have found that 2.6% of 245 differentially expressed genes contribute to regulating the inflammatory response. There are higher percentages of the differentially expressed genes in VAT than in SAT in prepubertal children [13] and adults [8], and the presence of inflammation and an immune response were the leading altered pathways in both studies. The observed discrepancy in the percentages might be explained by studies assessing differences in the gene expression between subcutaneous and visceral fat. 

Among the differentially expressed genes that were previously found in VAT from obese and normal-weight adults [11,12], we have replicated and validated by qPCR the down-regulation of the expression of the *CALCRL* (calcitonin receptor-like) gene reported by Baranova *et al.* [12] with a similar fold change (FC) value of approximately 2.5. Surprisingly, the results published by Gomez-Ambrosi *et al.* [11] describe genes that are differentially expressed in the opposite directions of our findings. The *CDKN1C* (*Cyclin-dependent kinase inhibitor 1C*), *NPR3* (*natriuretic peptide receptor C*) and *ADRB2* (*adrenergic beta-2-receptor surface*) genes were found to be down-regulated with FC values of 2.5, 3.2 and 2.2, respectively, which is in contrast to the up-regulation found in the present study. None of those three genes was validated by qPCR in the study performed in adults. By contrast, we found similar up-regulation of FC values for *NPR3* expression (4.31 and 5.1 by array and qPCR, respectively). This finding reinforces that validation of microarray data are required, as we discuss below.

### 2.3. Validation of Gene Expression Data by qPCR

To more precisely quantify the differential expression found by microarray analysis, a total of 73 genes (54 up- and 19 down-regulated) out of the total 245 differentially expressed genes, were selected for validation by qPCR. The criteria for gene selection were based on the degree of differential expression of each gene and its known functional roles in metabolic disease. qPCR assays were performed in 20 additional VAT samples (10 obese and 10 normal-weight samples). Table S3 depicts a comparison of the microarray and qPCR results. The differential gene expression data were only confirmed for 11 genes (20% of the total selected genes) (Table 2). All of these were up-regulated, except for *CALCRL*, which was down-regulated. Among the up-regulated genes, we validated the expression of leptin (*LEP*), one of the best-known genes, whose expression is altered in obesity, confirming the obesity-induced changes in mRNA expression. In accordance with the literature [17], we found elevated up-regulation of *LEP* mRNA expression in the VAT of obese children compared with lean subjects.

**Table 2:** Genes with altered expression, confirmed by qPCR, in the AT of obese prepubertal children compared to normal-weight children.

Symbol	Name	Microarrays		qPCR	
		Fold Change	*p* Value	Fold Change	* *p* Value
*CES1*	*carboxylesterase 1 *	11.74	0.001	3.0	0.050
*TNMD*	*tenomodulin protein*	7.19	0.028	5.5	0.028
*LEP*	*leptin (obesity homolog, mouse)*	4.44	0.005	3.5	0.050
*NPR3*	*natriuretic peptide receptor C*	4.31	0.005	5.1	0.006
*NQO1*	*NAD(P)H dehydrogenase, quinone 1*	3.99	0.006	2.8	0.041
*BHMT2*	*betaine-homocysteine methyltransferase 2*	3.14	0.005	2.3	0.049
*CRYAB*	*crystallin, alpha B*	2.25	0.005	2.6	0.034
*AFF1*	*AF4/FMR2 family, member 1*	2.20	0.004	2.9	0.041
*ANXA1*	*annexin A1*	2.14	0.037	1.9	0.049
*ITGB5*	*integrin, beta 5*	2.12	0.000	1.3	0.050
*CALCRL*	*calcitonin receptor-like*	-2.64	0.006	-2.4	0.034

* Δ*C*_t_ values were compared between obese and normal-weight children using the non-parametric Wilcoxon test (*p <* 0.05).

Interestingly, the expression (as delta *C*_t_) of several genes were correlated with some clinical and biochemical parameters (Table S4). Delta *C*_t_ represents the difference between the *C*_t_ value of a gene and that of the control gene (*GUS*). In other words, a higher delta *C*_t_ means a lower gene expression and conversely a lower delta *C*_t_ means a higher gene expression. It is interesting to note that delta *C*_t_ values of *ANXA1* (*r* = −0.55, *p* = 0.01), *NPR3 (r* = −0.54, *p* = 0.02) and *CALCRL* (*r* = −0.47, *p* = 0.04) were negatively associated with serum leptin levels. It is noteworthy that *TNMD* (*r* = −0.49, *p* *=* 0.03), *NPR3* (*r* = −0.56, *p* *=* 0.01)*,* and *AFF1* (*r* = −0.47, *p* *=* 0.05) were the only genes associated with BMI, but *TNMD* (*r* = −0.51, *p* *=* 0.02), *NPR3* (*r* = −0.48, *p* *=* 0.04) and *NQO1* (*r* = −0.45, *p* *=* 0.05) expression were associated with BMI z score. Similarly, *NPR-3* (*r* = −0.61, *p* *=* 0.01) and *AFF1* (*r* = −0.47, *p* *=* 0.04) were correlated with weight. Interestingly, delta *C*_t_ values of *CES1*, *TNMD*, *LEP*, *NPR3*, *NQO1*, *BHMT2* and *ITGB5* were negatively associated with systolic blood pressure. Meanwhile *ANXA1* were positively associated with serum HDL-C levels.

Microarray technology is a powerful tool for screening the gene expression profiles of biological samples, but it is mandatory to validate the obtained results with a highly reliable biotechnique that allows the precise quantification of the transcriptional abundance of the identified genes. qPCR is accepted as an appropriate method for gene expression array data validation. However, it is important to note that the study design followed by researchers widely differs at each stage of the validation process: (a) Gene selection is not always performed based on the degree of differential expression. Selection bias in genes, based on a pathway of interest, is frequent in genome-wide transcription analysis; (b) Separate samples should be used to perform an independent qPCR validation, whereas some studies use the same samples that were used in the array experiment. Considering these issues, we cannot compare and discuss the rate of validation of our study with those in previous studies.

Therefore, the discussion focuses on the genes whose expression, as determined by microarray, was validated by qPCR. Although different biological functions have been reported as mediated by a number of those genes, we have classified them according to the best-known function. 

#### 2.3.1. Lipid and Amino Acid Metabolism 

A key factor in energy balance is the mobilization of lipids through lipolysis in fat cells, and basal lipolysis is higher in obese individuals than non-obese individuals [18]. The main enzymes mediating the lipolysis in AT are adipose triglyceride lipase and hormone-sensitive lipase; however, the importance of other lipases, such as carboxylesterase 1 (CES1), is unclear and CES1 has been reported to play a major role in the trafficking of lipids and lipid metabolism in hepatocytes [19]. We found up-regulation of *CES1* expression; this gene encodes an enzyme that mediates adipocyte lipolysis through the hydrolysis of cholesteryl esters and triglycerides [18]. The expression of *CES1* is highly regulated in adult AT; there are higher levels in obese subjects and lower levels during a weight loss period [20]. In addition, the SAT mRNA levels of *CES1* are positively associated with BMI, HOMA, fasting glucose, insulin and TAG in adults [21,22]. Our findings in the VAT from children confirm the findings in adults, indicating that *CES1* may have a role in the development of obesity-associated phenotypes. It is not yet known whether this lipase is more important to the TAG metabolism in the liver than to AT.

In contrast to the increased AT lipolytic activity, we found up-regulation of *NPR3*, which may have an antilipolytic effect through receptor inhibition of lipolysis mediated by natriuretic peptides [23]. The results from a large-cohort epidemiological study suggested that in the obese state, insulin might suppress the circulating levels of natriuretic peptides via up-regulating NPR-3 expression [24]. However, it is noteworthy that the activation of NPR-3 in human thyroid cells increased the cAMP levels, which may suggest an alternative role for NPR-3 [25] in AT. 

The plasma concentrations of several amino acids are elevated in obesity and insulin resistance in humans, but there is no conclusive evidence about whether these amino acid alterations are causal. In the present study, we found, for the first time, up-regulated expression of the *betaine-homocysteine methyltransferase 2* (*BHMT2*) gene. BHMT2 catalyses the formation of the amino acid methionine from homocysteine using the choline metabolite betaine as a methyl donor. Although this enzyme is predominantly found in the liver, deletion of the mouse *Bhmt* gene reduces adiposity owing to an increased whole body metabolic rate, impaired TAG synthesis, and enhanced glucose oxidation in white AT [26]. This highlights the relationship between the over-expression of *BHMT2* and development of obesity. 

#### 2.3.2. Oxidative Stress and Extracellular Matrix Related Genes

AT function is regulated by the physiological interaction between cells and a variety of extracellular proteins [27]. Among them, *integrin beta 5* (*ITGB5*) was up-regulated in our sample of obese children. Henegar *et al.* [28] also showed that *ITGB5* and other genes encoding the members of the integrin family were significantly induced and co-expressed in SAT from obese adults. Moreover, *tenomodulin* (*TNMD*), an angiogenesis inhibitor, which may play a role in AT function and has been implicated in extracellular matrix remodelling [29,30], was up-regulated in our sample of obese children. This finding is in concordance with the results of Saiki *et al.* [30]. In addition, the *TNMD* mRNA levels strongly correlate with the BMI, insulin sensitivity and leptin levels in adults [29,30]. 

Increased oxidative stress, resulting in the generation of reactive oxygen species (ROS), is observed in obesity, which might explain the over-expression of *NAD(P)H dehydrogenase quinone 1 (NQO1)* in the present study. *NQO1* is highly expressed in human AT, especially in large adipocytes, and it correlates with adiposity, glucose tolerance, and liver dysfunction markers in obese adults [31].

#### 2.3.3. Adipogenesis Related Genes

We found up-regulation of *CRYAB* (*crystallin*, *alpha B*), which has been implicated in apoptosis, and *CRYAB* expression, induced during adipogenesis. In agreement with our results, Lehr *et al.* demonstrated that *CRYAB* expression in the VAT of obese subjects is significantly higher than in that of lean controls [32]. In addition, *calcitonin receptor-like* (*CALCRL*), a G-protein coupled receptor for the adrenomedullin peptide, was found to be down-regulated. Adrenomedullin is present at higher levels in both the plasma and AT of obese individuals than in those of lean subjects and is considered to be a member of the adipokine family [33]. Adrenomedullin has been described as an antiadipogenic factor because the reduction of adrenomedullin synthesis strongly accelerates adipose differentiation [34]. The down-regulation of the receptor found in children in the present study, and in adults by Baranova *et al.* [12] could explain the increased levels of adrenomedullin described in obesity.

Another adipogenesis-associated gene with up-regulated expression was the *AF4/FMR2 family member 1* (*AFF1*). Although a polymorphism in the *AFF1* gene has been associated with an increase in circulating TAG, its function in lipid metabolism is not known [35]. *AFF1* may have a role in regulating the cyclin-dependent kinase inhibitor CDKN1B [36] and may therefore be involved in cell cycle regulation. Genes of the cyclin-dependent kinase inhibitor family are involved in cell proliferation and differentiation within AT [37] and could therefore be involved in the hyperplasia mechanisms at the onset of obesity.

#### 2.3.4. Inflammation-Related Gene

Validation of the microarray data confirmed the up-regulation of *Annexin A1* (*ANXA1*), a protein that mediates the anti-inflammatory action of glucocorticoids. ANXA1 is a member of the annexin superfamily of calcium-binding and phospholipid-binding proteins; these results in children are in agreement with previous results in obese subjects after weight loss [28]. It is interesting that the plasma levels of ANXA1 in humans are inversely correlated with markers of adiposity, although *ANXA1* gene and protein expression are significantly up-regulated during adipogenesis in a human adipocyte cell line [38].

## 3. Experimental Section 

### 3.1. Study Population

We recruited 31 Caucasian children, aged 6–12 years, undergoing abdominal surgery in the Pediatric Surgery Unit of the Hospital Reina Sofía (Cordoba, Spain) with a diagnosis of hernia or false positive diagnosis of acute appendicitis. Of 31 children, 15 (13 boys and 2 girls) were obese, and 16 were normal-weight children (11 boys and 5 girls) according to Cole *et al.* [39]. All children were in the prepubertal period based on the Tanner criteria (Tanner I) (Tanner, 1962) and as validated by plasma sex hormone concentrations. The inclusion criteria were a good state of health, age between 5 and 14 years and the absence of endogenous obesity. Exclusion criteria were disease or malnutrition and the use of a medication that alters blood pressure or glucose or lipid metabolism. 

The children’s parents or guardians were informed about the purpose and procedures of the study before written consent was obtained, and all children agreed to participate. The protocol was performed in accordance with the Revised Declaration of Helsinki and following the recommendations of the Good Clinical Practice of the European Union (document 111/3976/88 July 1990) and legal in-force Spanish regulations, which regulate clinical investigation in human beings (RD 223/04 about Clinical Assay), as approved by the Ethics Committees of all participants’ institutions.

### 3.2. Anthropometric and Biochemical Measurements

A single examiner performed all anthropometric measurements according to standardized methods. Body weight (kg) and height (cm) were measured using standardized procedures, and BMI was calculated as the weight (kg) divided by the square root of the height (m^2^). Systolic and diastolic blood pressures were measured three times at 5-min intervals while children were seated, using a standardized mercury sphygmomanometer and adapted cuff size; the final two measurements were averaged for the analysis. The pubertal stage was determined in each patient according to Tanner’s criteria and was validated by plasma sex hormone concentrations. Children in Tanner stage I were considered prepubertal, and those in Tanner stages II–V were considered pubertal.

Blood was obtained by venipuncture before surgery and was divided into aliquots for immediate analysis or storage at −80 °C until analysis. Glucose was analysed using the glucose oxidase method in an automatic analyser (CV 1%) (Roche-Hitachi Modular PyD Autoanalyzer, Roche Laboratory Systems, Mannheim, Germany), and plasma insulin was analysed by radioimmunoassay (CV 2.6%) (Axsym, Abbott Laboratories, Chicago, IL, USA). The insulin resistance index was calculated with the homeostatic assessment model (HOMA-IR). Total adiponectin was determined by RIA (LINCO Research Inc) and leptin by ELISA (BioSource International Inc., Camarillo, CA, USA). The plasma total, HDL and LDL cholesterol; TAG; apolipoprotein A-1 and –B; and C-reactive protein (CRP) levels were analysed using an automatic analyser (Roche-Hitachi Modular P and D Autoanalyzer). 

### 3.3. Adipose Tissue Biopsy and Total RNA Preparation

During abdominal surgery, approximately 400 mg of visceral (intra-abdominal) AT was collected and excised into pieces of approximately 150 mg, which were each immediately immersed in 1.5 mL RNA-later solution (Qiagen, Izasa, Spain) and stored at −80 °C until analysis. Total RNA from VAT biopsies was isolated using the RNeasy Lipid Tissue Midi Kit (Qiagen, Izasa, Spain). The integrity of the total RNA was checked by visual inspection of the 28S and 18S RNA on agarose gel electrophoresis.

### 3.4. Microarray Analysis

RNA samples from six control and five obese children were used for the microarray analysis. A total of 11 chips were used (one per tissue sample). Subsequent RNA processing procedures followed the protocols in the GeneChip Expression Analysis Technical Manual (Affymetrix, Santa Clara, CA, USA). Briefly, five micrograms of total RNA was used as the starting material for the Affymetrix sample preparation. cDNA was synthesized with the SuperScript Choice System Kit (Life Technologies Corporation, Izasa, Spain). The cDNA was used for the cRNA synthesis using an IVT Labeling kit (Affymetrix, Santa Clara, CA, USA) and then purified using a GeneChip Sample Cleanup Module Kit (Affymetrix). Fifteen micrograms of biotin-labelled cRNA was fragmented and hybridized to the HG-U133 Plus2.0 array (Affymetrix), which offers comprehensive analysis of the genome-wide expression on a single array, analysing the expression level of over 47,200 transcripts corresponding to 38,500 well-characterized human genes.

After scanning, the digitized image data were processed using GCOS 1.1 software (Affymetrix), and analysed with GeneSpring software (Silicongenetics, Fremont, CA, USA). Significant differences between the obese and control genes were tested using a one-way ANOVA. Hierarchical clustering analysis was performed with Pearson correlation for sample classification. Differences were considered significant at *p* < 0.05. The results are expressed as the FC of obese *vs.* control children.

Among the differentially expressed genes (*p* < 0.05 and FC ≥ 2 or FC ≤ 2), dataset functional analysis was performed using Gene Ontology (GO) terms with the Database for Annotation, Visualization and Integrated Discovery (DAVID), available online [40]. Additionally, biological networks and canonical pathways were analyzed by QIAGEN’s Ingenuity^®^ Pathway Analysis, available online [41], for the 245 genes with an altered expression. Both direct and indirect relationships were considered. The association between the data set and the canonical pathway was determined by a P value calculated with the right-tailed Fisher exact test. The complete data sets in the present study comply with the MIAME (Minimum Information About a Microarray Experiment) requirements, which are available from the National Center for Biotechnology Information Gene Expression Omnibus web site [42]; accession no. GSE9624.

### 3.5. qPCR Analysis

To validate the differential gene expression obtained by microarrays, qPCR was performed on a panel of 73 selected genes based on their possible implication in the development of obesity and microarray differential expression (*p* < 0.05 and FC ≥ 2 or FC ≤ 2). The validation was performed on 20 additional VAT samples (10 obese and 10 control samples). Total RNA was obtained as described above. After DNase I treatment (Ambion, Paisley, UK), 1 μg of total RNA was reverse transcribed into cDNA using the High-Capacity cDNA Archive Kit (Applied Biosystems, Foster City, CA, USA). qPCR was performed using a GeneAmp 7900 Sequence Detection System and TaqMan Assays-on-Demand Gene Expression Products (Applied Biosystems). The TaqMan Human Endogenous Control Plate (Applied Biosystems) was used to select the best candidate as an endogenous control gene (*GUS*). The ΔΔ*C*_t_ method was used to calculate the relative changes in the mRNA abundance as FC values.

### 3.6. Statistical Analysis

Demographic and biochemical data were compared between obese and normal weight children using the non-parametric Mann-Whitney U test.

Statistical analysis of qPCR data was performed by analysing the Δ*C*_t_ values, which were calculated by subtracting the *C*_t_ number of each target gene from that of the endogenous control *GUS* gene. The Δ*C*_t_ values were compared between obese and control children with the non-parametric Wilcoxon test, as recommended by Yuan *et al.* [43]. Spearman correlation coefficient was performed between gene expression values, as delta *C*_t_, and clinical and biochemical characteristics. Statistical analysis was carried out using the SPSS 22.0 software for Windows (SPSS Inc., Chicago, IL, USA).

## 4. Conclusions

To the best of our knowledge, this study reports the first investigation of prepubertal children in which obesity-induced gene expression changes have been globally analysed in VAT using genome wide microarrays, providing valuable new insight into the pathology of early-onset human obesity. Our data demonstrate that the VAT of obese children exhibits a gene expression profile that differs from their normal-weight peers. More genes and a wider spectrum of genes were expressed in the AT of obese children, including some genes that have not previously been described. Differential gene expression that was validated by qPCR confirmed the up-regulation of genes related to lipid and amino acid metabolism (*CES1*, *NPRR3* and *BHMT2*), oxidative stress and extracellular matrix regulation (*TNMD* and *NQO1*), adipogenesis (*CRYAB* and *AFF1*) and inflammation (*ANXA1*), while *CALCRL* gene expression was the only gene confirmed to be downregulated. 

In conclusion, this study in prepubertal children demonstrates that there is a different gene expression signature in the VAT of obese pre-pubertal children, some of them previously proposed to influence the pathogenesis of adulthood obesity, as well as newly uncovered dysregulated genes that may be candidate genes in the aetiology of obesity. Our findings reinforce the current concept that obesity is a complex trait and provides new insight into the pathology of early-onset human obesity. However, our study has some limitations; we had a relatively low number of subjects, but also we used VAT, which is composed of adipocytes, together with other cells, such as macrophages, vascular cells and other blood cells. Further studies are needed to clarify the specific role of these genes in childhood obesity.

## Supplementary Materials

Supplementary materials can be found at http://www.mdpi.com/1422-0067/16/04/7723/s1.

## References

[1] De Bruyne R.M., Fitzpatrick E., Dhawan A. (2010). Fatty liver disease in children: Eat now pay later. Hepatol. Int..

[2] Alligier M., Meugnier E., Debard C., Lambert-Porcheron S., Chanseaume E., Sothier M., Loizon E., Hssain A.A., Brozek J., Scoazec J.Y. (2012). Subcutaneous adipose tissue remodeling during the initial phase of weight gain induced by overfeeding in humans. J. Clin. Endocrinol. Metab..

[3] Kershaw EE, Flier JS. (2004). Adipose tissue as an endocrine organ. J. Clin. Endocrinol. Metab..

[4] Tchernof A., Despres J.P. (2013). Pathophysiology of human visceral obesity: An update. Physiol. Rev..

[5] Ibrahim M.M. (2010). Subcutaneous and visceral adipose tissue: Structural and functional differences. Obes. Rev..

[6] Insenser M., Montes-Nieto R., Vilarrasa N., Lecube A., Simó R., Vendrell J., Escobar-Morreale H.F. (2012). A nontargeted proteomic approach to the study of visceral and subcutaneous adipose tissue in human obesity. Mol. Cell. Endocrinol..

[7] Gil A., Olza J., Gil-Campos M., Gomez-Llorente C., Aguilera C.M. (2011). Is adipose tissue metabolically different at different sites?. Int. J. Pediatr. Obes..

[8] Gerhard G.S., Styer A.M., Strodel W.E., Roesch S.L., Yavorek A., Carey D.J., Wood G.C., Petrick A.T., Gabrielsen J., Ibele A. (2014). Gene expression profiling in subcutaneous, visceral and epigastric adipose tissue of patients with extreme obesity. Int. J. Obes..

[9] Linder K., Arner P., Flores-Morales A., Tollet-Egnell P., Norstedt G. (2004). Differentially expressed genes in visceral or subcutaneous adipose tissue of obese men and women. J. Lipid Res..

[10] Lee Y.H., Nair S., Rousseau E., Allison D.B., Page G.P., Tatarami P.A., Bogardus C., Permana P.A. (2005). Microarray profiling of isolated abdominal subcutaneous adipocytes from obese *vs.* non-obese Pima Indians: Increased expression of inflammation-related genes. Diabetologia.

[11] Gomez-Ambrosi J., Catalán V., Diez-Caballero A., Martinez-Cruz L.A., Gil M.J., García-Foncillas J., Cienfuegos J.A., Salvador J., Mato J.M., Frühbeck G. (2004). Gene expression profile of omental adipose tissue in human obesity. FASEB J..

[12] Baranova A., Collantes R., Gowder S.J., Elariny H., Schlauch K., Younoszai A., King S., Randhawa M., Pusulury S., Alsheddi T. (2005). Obesity-related differential gene expression in the visceral adipose tissue. Obes. Surg..

[13] Tam C.S., Heilbronn L.K., Henegar C., Wong M., Cowell C.T., Cowley M.J., Kaplan W., Clément K., Baur L.A. (2011). An early inflammatory gene profile in visceral adipose tissue in children. Int. J. Pediatr. Obes..

[14] Ibáñez L., de Zegher F. (2006). Puberty and prenatal growth. Mol. Cell. Endocrinol..

[15] Olza J., Aguilera C.M., Gil-Campos M., Leis R., Bueno G., Martínez-Jiménez M.D., Valle M., Cañete R., Tojo R., Moreno L.A. (2012). Myeloperoxidase is an early biomarker of inflammation and cardiovascular risk in prepubertal obese children. Diabetes Care.

[16] Olza J., Aguilera C.M., Gil-Campos M., Leis R., Bueno G., Valle M., Cañete R., Tojo R., Moreno L.A., Gil A. (2014). Waist-to-height ratio, inflammation and CVD risk in obese children. Public Health Nutr..

[17] Lönngvist F., Arne P., Nordfors L., Schalling M. (1995). Overexpression of the obese (ob) gene in adipose tissue of human obese subjects. Nat. Med..

[18] Arner P., Langin D. (2007). The role of neutral lipases in human adipose tissue lipolysis. Curr. Opin. Lipidol..

[19] Blais D.R., Lyn R.K., Joyce M.A., Rouleau Y., Steenbergen R. (2010). Activity based protein profiling identifies a host enzyme, carboxylesterase 1, which is differentially active during hepatitis C virus replication. J. Biol. Chem..

[20] Jernas M., Olsson B., Arner P., Jacobson P., Sjöström W.A, Froguel P., McTernan P.G., Hoffstedt J., Carlsson L.M.S. (2009). Regulation of carboxylesterase 1 (CES1) in human adipose tissue. Biochem. Biophys. Res. Commun..

[21] Nagashima S., Yagyu H., Takahashi N., Kurashina T., Takahashi M., Tsuchita T., Tazoe F., Wang X.L., Bayasgalan T., Sato N. (2011). Depot-specific expression of lipolytic genes in human adipose tissues: Association among CES1 expression, triglyceride lipase activity and adiposity. J. Atheroscler. Thromb..

[22] Friedrichsen M., Poulsen P., Wojtaszewski J., Hansen P.R., Vaag A., Rasmussen H.B. (2013). Carboxylesterase 1 gene duplication and mRNA expression in adipose tissue are linked to obesity and metabolic function. PLoS ONE.

[23] Lafontan M., Moro C., Berlan M., Crampes F., Sengenes C., Galitzky J. (2008). Control of lipolysis by natriuretic peptides and cyclic GMP. Trends Endocrinol. Metab..

[24] Pivovarova O., Gögebakan Ö., Kloöting N., Sparwasser A., Weickert M.O., Haddad I., Nikiforova V.J., Bergmann A., Kruse M., Seltmann A.C. (2012). Insulin up-regulates natriuretic peptide clearance receptor expression in the subcutaneous fat depot in obese sunjects: A missing link between CVD risk and obesity?. J. Clin. Endocrinol. Metab..

[25] Selleti D.F., Perrella G., Doi S.Q., Curcio F. (2001). Natriuretic peptides increase cAMP production in human thyrocytes via the natriuretic peptide clearance (NPR-C). Regul. Pept..

[26] Teng Y.W., Ellis J.M., Coleman R.A., Zeisel S.H. (2012). Mouse betaine-homocysteine *S*-methyltransferase deficiency reduces body fat via increasing energy expenditure and impairing lipid synthesis and enhancing glucose oxidation in white adipose tissue. J. Biol. Chem..

[27] Chun T.H. (2012). Peri-adipocyte ECM remodeling in obesity and adipose tissue fibrosis. Adipocyte.

[28] Henegar C., Tordjman J., Achard V., Lacasa D., Cremer I., Guerre-Millo M., Poitou C., Basdevant A., Stich V., Viguerrie N. (2008). Adipose tissue transcriptomic signature highlights the pathological relevance of extracelular matrix in human obesity. Genome Biol..

[29] Kolehmainen M., Salopuro T., Schwab U.S., Kekalainen J., Kallio P., Laaksonen D.E., Pulkkinen L., Lindi V.I., Sivenius K., Mager U. (2008). Weight reduction modulates expression of genes involved in extracellular matrix and cell death: The GENOBIN study. Int. J. Obes..

[30] Saiki A., Olsson M., Jernas M., Gummesson A., Mcternan PG., Andersson J., Jacobson P., Sjöholm K., Olsson P., Yamamura S. (2009). Tenomodulin is highly expressed in adipose tissue, increased in obesity, and down-regulated during diet-induced weight loss. J. Clin. Endocrinol. Metab..

[31] Palming J., Sjöholm K., Jernas M., Lustig T.C., Gummesson A., Romeo S., Lönn L., Lönn M., Carlsson B., Carlsson L.M. (2007). The expression of NAD(P)H: Quinine oxidoreductase 1 is high in human adipose tissue, reduced by weight loss, and correlates with adipostity, insulin sensitivity, and markers of liver dysfunction. J. Clin. Endocrinol. Metab..

[32] Lehr S., Hartwig S., Lamers D., Famulla S., Müller S., Hanisch F.G., Cuvelier C., Ruige J., Eckardt K., Ouwens M. (2012). Identification and validation of novel adipokines released from primary human adipocytes. Mol. Cell. Proteomics.

[33] Li Y., Jiang C., Wang X., Zhang Y., Shibahara S., Takahashi K. (2007). Adrenomedullin is a novel adipokine: Adrenomedullin in adipocytes and adipose tissues. Peptides.

[34] Harmancey R., Senard J.M., Rouet P., Pathak A., Smih F. (2007). Adrenomedullin inhibits adipogenesis under transcriptional control of insulin. Diabetes.

[35] Waterworth D.M., Ricketts S.L., Song K., Chen L., Zhao J.H., Ripatti S., Aulchenko Y.S., Zhang W., Yuan X., Lim N. (2010). Genetic variants influencing circulating lipids levels and risk of coronary artery disease. Arterioscler. Thromb. Vasc. Biol..

[36] Xia Z.B., Popovic R., Chen J., Theisler C., Stuart T., Santillan D.A., Erfurth F., Diaz M.O., Zeleznik-Le N.J. (2005). The MLL fusion gene, MLL-AF4, regulates cyclin-dependent kinase inhibitor CDKN1B (p27kip1) expression. Proc. Natl. Acad. Sci. USA.

[37] Naaz A., Holsberger D.R., Iwamoto G.A., Nelson A., Kiyokawa H., Cooke P.S. (2004). Loss of cyclin-dependent kinase inhibitors produces adipocyte hyperplasia and obesity. FASEB J..

[38] Kosicka A., Cunliffe A.D, Mackenzie R., Zariwala M.G., Parretti M., Flower R.J., Renshaw D. (2013). Attenuation of plasma annexin A1 in human obesity. FASEB J..

[39] Cole T.J., Bellizi M.C., FLegal K.M., Dietz W.H. (2000). Establishing a standard definition for child overweight and obesity worldwide: International survey. BMJ.

[40] Database for Annotation, Visualization and Integrated Discovery (DAVID). http://david.abcc.ncifcrf.gov/.

[41] QIAGEN’s Ingenuity® Pathway Analysis. www.qiagen.com/ingenuity.

[42] National Center for Biotechnology Information Gene Expression Omnibus web site. http://www.ncbi.nlm.nih.gov/projects/geo/.

[43] Yuan J.S., Reed A., Chen F.L., Stewart N.C. (2006). Statistical analysis of real-time PCR data. BMC Bioinform..

