# Supplementary material for: Genome-Wide Expression in Visceral Adipose Tissue from Obese Prepubertal Children
Source: Int J Mol Sci. 2015 Apr 8;16(4):7723–37. doi: 10.3390/ijms16047723 (PMC4425045; doi:10.3390/ijms16047723)
Supplement: Supplementary file 1 [file ijms-16-07723-s001.zip › ijms-79084-Supplementary Information/Figure S1.pdf]

Supplementary Information

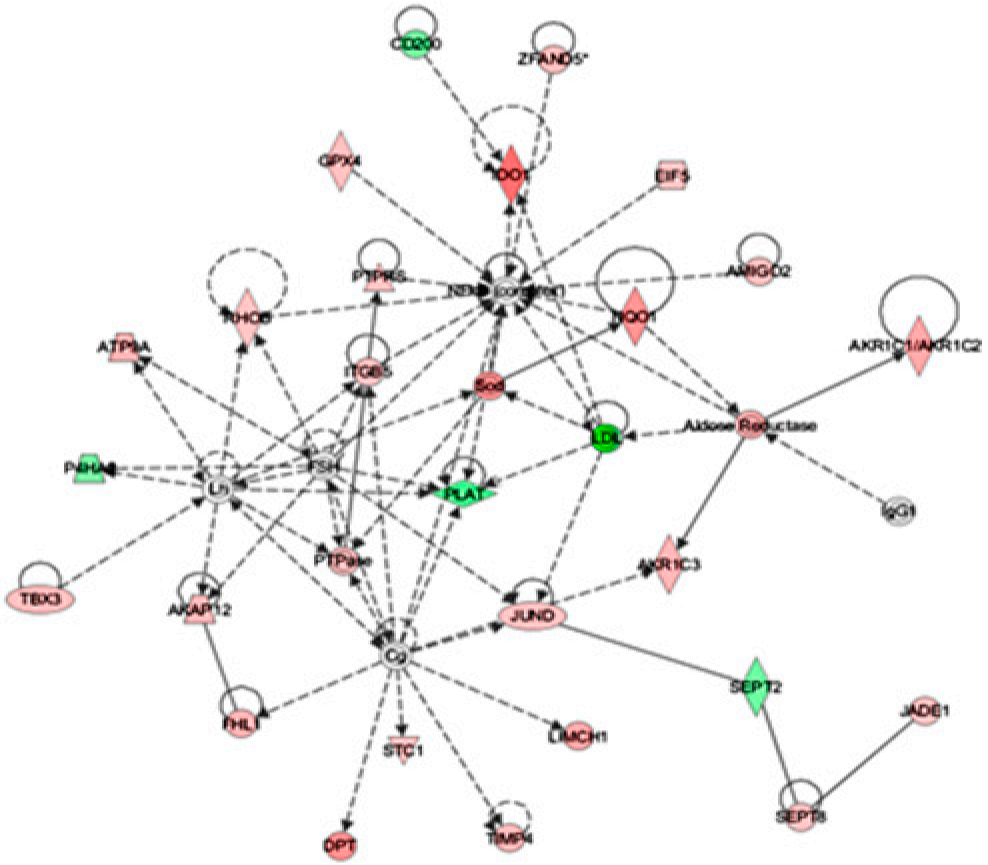

Figure S1. Funtional network analysis by Ingenuity Pathway Analysis (IPA). The top scoring network (Score of 26 and 35 focus molecules) generated by Ingenuity Pathway Analysis (IPA) under the function “Lipid Metabolism, Small Molecule Biochemistry, Endocrine System Development and Function”. Genes upregulated are in red and in green genes downregulated.
